# Classification and Diagnosis of Residual Thyroid Tissue in SPECT Images Based on Fine-Tuning Deep Convolutional Neural Network

**DOI:** 10.3389/fonc.2021.762643

**Published:** 2021-10-28

**Authors:** Yinxiang Guo, Jianing Xu, Xiangzhi Li, Lin Zheng, Wei Pan, Meiting Qiu, Shuyi Mao, Dongfei Huang, Xiaobo Yang

**Affiliations:** ^1^ Guangxi Key Laboratory on Precise Prevention and Treatment for Thyroid Tumor, The Second Affiliated Hospital, Guangxi University of Science and Technology, Liuzhou, China; ^2^ School of Science, Guangxi University of Science and Technology, Liuzhou, China; ^3^ Department of Public Health, School of Medicine, Guangxi University of Science and Technology, Liuzhou, China; ^4^ School of Microelectronics and Materials Engineering, Guangxi University of Science and Technology, Liuzhou, China; ^5^ Department of Nuclear Medicine, The Second Affiliated Hospital of Guangxi University of Science and Technology, Liuzhou, China

**Keywords:** SPECT image, thyroid cancer, deep learning, fine-tuning, classification diagnosis, overtreatment

## Abstract

Patients with thyroid cancer will take a small dose of ^131^I after undergoing a total thyroidectomy. Single-photon emission computed tomography (SPECT) is used to diagnose whether thyroid tissue remains in the body. However, it is difficult for human eyes to observe the specificity of SPECT images in different categories, and it is difficult for doctors to accurately diagnose the residual thyroid tissue in patients based on SPECT images. At present, the research on the classification of thyroid tissue residues after thyroidectomy is still in a blank state. This paper proposes a ResNet-18 fine-tuning method based on the convolutional neural network model. First, preprocess the SPECT images to improve the image quality and remove background interference. Secondly, use the preprocessed image samples to fine-tune the pretrained ResNet-18 model to obtain better features and finally use the Softmax classifier to diagnose the residual thyroid tissue. The method has been tested on SPECT images of 446 patients collected by local hospital and compared with the widely used lightweight network SqueezeNet model and ShuffleNetV2 model. Due to the small data set, this paper conducted 10 random grouping experiments. Each experiment divided the data set into training set and test set at a ratio of 3:1. The accuracy and sensitivity rates of the model proposed in this paper are 96.69% and 94.75%, which are significantly higher than other models (p < 0.05). The specificity and precision rates are 99.6% and 99.96%, respectively, and there is no significant difference compared with other models. (p > 0.05). The area under the curve of the proposed model, SqueezeNet, and ShuffleNetv2 are 0.988 (95% CI, 0.941–1.000), 0.898 (95% CI, 0.819–0.951) (p = 0.0257), and 0.885 (95% CI, 0.803–0.941) (p = 0.0057) (p < 0.05). We prove that this thyroid tissue residue classification system can be used as a computer-aided diagnosis method to effectively improve the diagnostic accuracy of thyroid tissue residues. While more accurately diagnosing patients with residual thyroid tissue in the body, we try our best to avoid the occurrence of overtreatment, which reflects its potential clinical application value.

## 1 Introduction

Thyroid nodules are irregular masses around the thyroid gland and are common diseases of the endocrine system. Thyroid nodules can be divided into benign and malignant. According to the global cancer statistics report, the incidence of malignant thyroid nodules, that is, thyroid cancer, ranks first in endocrine malignant tumors. Total thyroidectomy is the most commonly used clinical surgery to treat thyroid cancer. Single-photon emission computed tomography (SPECT) is one of the representative techniques of nuclear medicine imaging (1). Compared with general medical imaging technology, SPECT can not only display the morphology and structure of tissues and organs, but more importantly, it can provide some characteristic information of tissues and cells at the molecular level, thereby showing the metabolic status of tissues and organs. In 2020, the total number of single-photon imaging inspections in China was more than 2.5 million (2). At this stage, it is mainly based on SPECT images to determine whether there is thyroid tissue remaining in the body after ablation of thyroid cancer patients. During the ablation treatment of thyroid cancer patients, SPECT images can dynamically observe the effect before and after treatment and the metastasis of the lesion from the pathological point of view (3). Therefore, SPECT is widely used in clinical diagnosis in ^131^I ablative treatment with its unique advantages (4, 5).

In 2015, the “Guidelines for the Diagnosis and Treatment of Adult Thyroid Nodules and Differentiated Thyroid Cancer” issued by the American Thyroid Association (ATA) pointed out that in order to reduce unnecessary radiation damage and avoid overtreatment problems, it is recommended to use 30 mCi for ^131^I ablative treatment for middle and low-risk patients (6). In clinical practice, 2–5 mCi is generally used for the detection of residual thyroid tissue after total thyroidectomy (7). Because of the dose of 30 mCi which is close to the dose for one treatment purpose and is not used for diagnosis, which may cause patients to develop iodine resistance, doctors use 5 mCi instead of 30 mCi for ^131^I when diagnosing whether thyroid tissue remains. Due to the interference of noise caused by SPECT’s own imaging characteristics, equipment environment, and patient’s condition, there are defects such as insignificant specificity of manual observation pictures, large basic dosage of ^131^I, long diagnosis time, low accuracy, and inconsistent standards, which lead to insufficient treatment pertinence, greatly increasing the blindness of treatment and the suffering of patients.

In the field of computer-aided diagnosis, the classification and prediction of thyroid tissue residues after ablation is still at a blank stage, but previous studies have confirmed that computer-aided diagnosis technology based on medical images is an efficient method to improve diagnosis accuracy. Therefore, the goal of this paper is to design a computer-aided diagnosis system based on the deep convolutional neural network (DCNN) (8) to improve the accuracy of classification of thyroid tissue residues, aiming to verify the clinical role of the CAD system in the diagnosis of thyroid tissue residues.

## 2 Materials and Methods

### 2.1 Database

All experimental protocols in this study were approved by the Second Affiliated Hospital of Guangxi University of Science and Technology. We collected a total of 446 SPECT images of thyroid cancer patients who received a low-dose ^131^I diagnosis after total thyroidectomy. Among them, there were 346 SPECT images with residual thyroid tissue in the body and 100 SPECT images with no residual thyroid tissue in the body. The final diagnosis was given by two nuclear medicine doctors with more than 10 years of clinical diagnosis experience. In addition, all patients who were diagnosed with residual thyroid tissue in the body will receive a treatment dose of ^131^I treatment, and the residual thyroid tissue can be clearly observed under treatment ^131^I SPECT imaging. All patients who were diagnosed as having no residual thyroid tissue in the body will undergo a blood test for thyroid function, and all indicators are normal. Therefore, the accuracy of the label is guaranteed.

We refer to the ATA guidelines for the classification of high-, medium-, and low-risk recurrence populations and use the pathological results of the study subjects as the gold standard to count the number of high-, medium-, and low-risk recurrence populations in this study. Among them, 283 (63.4%) patients were at a moderate risk of recurrence, 139 (31.2%) patients were at a low risk of recurrence, and 24 (5.4%) patients were at a high risk of recurrence. Generally speaking, people with low-risk recurrence risk are less likely to have cancer recurrence. People with high-risk recurrence risk can judge whether thyroid tissue remains from the level of thyroglobulin. In order to improve the generalization ability and applicability of the model, we adopted all the images as experimental objects. The plane images of the neck collected in this study were taken by Optima NM/CT 640 machine (GE Healthcare). The image acquisition protocol uses a high-energy, parallel-hole collimator, with an energy peak of 364 keV, a window width of 20%, a matrix of 256 × 256, 5~10 minutes/frame, and an imaging time of about 1 h; the planar ^131^I neck imaging was completed in both anterior and posterior projections. **Table 1** shows the characteristics of the research objects.

**Table 1:** Characteristics of study subjects.

Parameter	Value
Mean age (years)	41.8 ± 11.4
Range	13∼79
**Patient gender, n (%)**	87 (19.5%)
Male	
Female	359 (80.5%)
**Pathology, n (%)**	438 (98.2%)
Papillary thyroid carcinoma	
Follicular thyroid carcinoma	5 (1.1%)
Medullary thyroid carcinoma	0 (0%)
Undifferentiated thyroid carcinoma	3 (0.7%)

### 2.2 Image Processing

The sensitivity of the SPECT image itself is relatively low and is affected by attenuation and scattering. There are background ghosts and edge artifacts in the image, which affect the accurate judgment of the disease. The main purpose of SPECT image processing is to remove noise and edge environment interference through digital image processing technology to accurately segment the thyroid bed area from the image. This paper uses python as the programming language and processes images based on the OpenCV open-source library.

#### 2.2.1 Histogram Equalization

**Figure 1:**
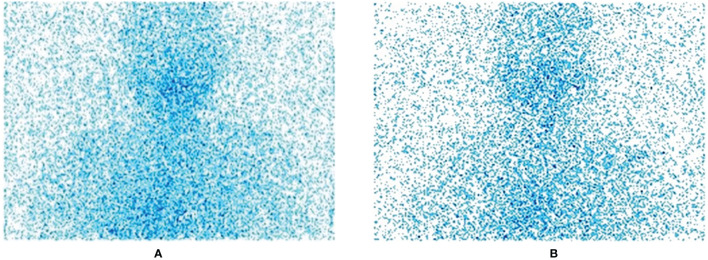
**(A)** the original SPECT image with residue; **(B)** the original SPECT image without residue.

The images we collected are all standard medical images in DICOM format. In order to facilitate preprocessing and network training, we first convert them to JPG format images, centering on the thyroid bed area, and crop all image sizes to 800 × 750 size; this step is to remove irrelevant parts and information artifacts in the image. **Figure 1** shows the original SPECT image without residue (A) and the original SPECT image with residue (B).

Histogram equalization is a commonly used gray-scale enhancement algorithm (9). It converts an image into an image with a uniform number of pixels in each gray level through gray-scale transformation. At this time, the entropy of the image is the largest, and the amount of information it contains is also the largest. If the grayscale histogram of an image almost covers the entire grayscale value range, and the number of individual grayscale values is more prominent, and the entire grayscale value distribution is approximately uniform, then this image has a larger dynamic range of gray scale and higher contrast, and the details of the image are more abundant.

**Figure 2:**
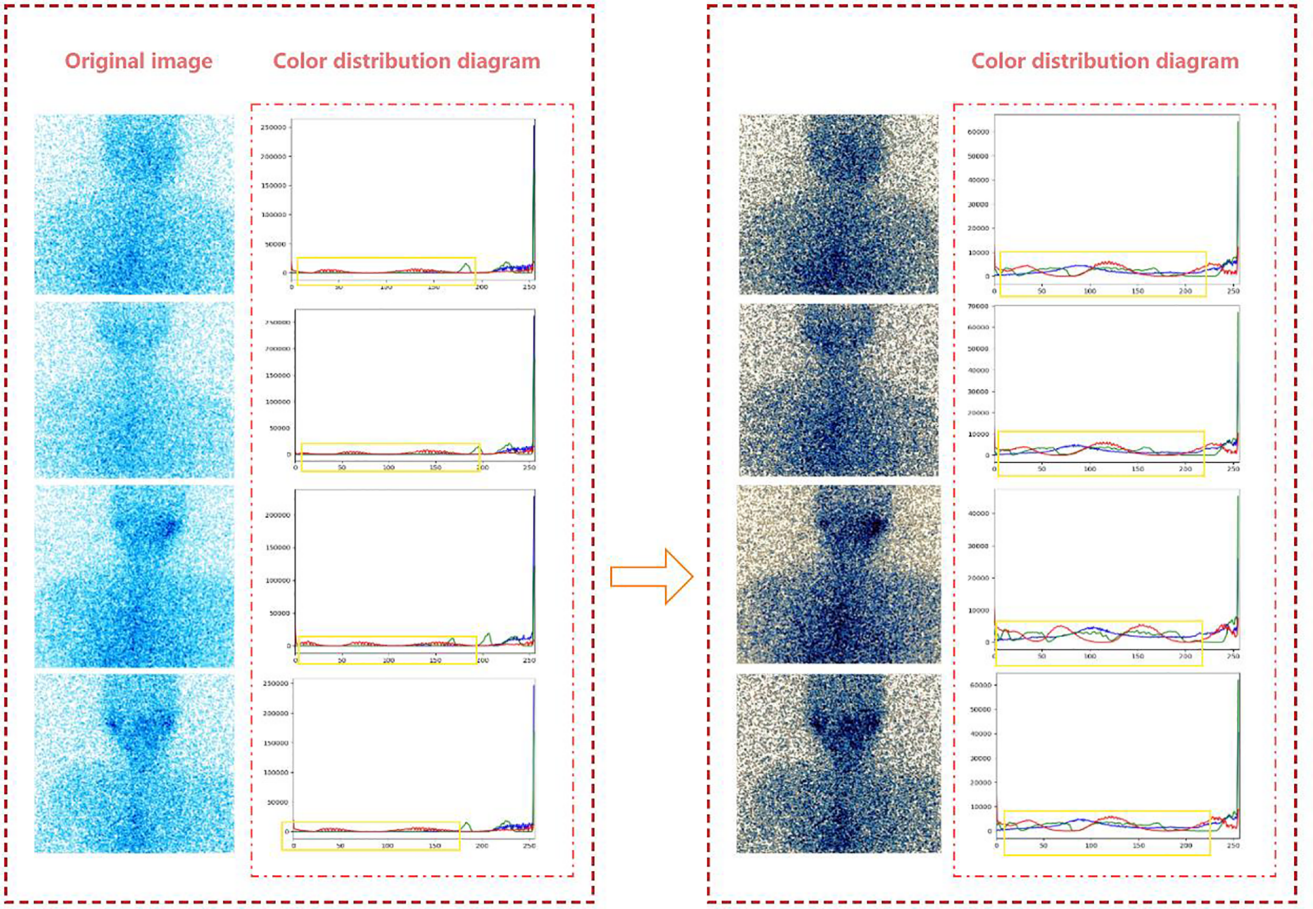
The effect of histogram equalization on the improvement of SPECT image quality and the RGB.

SPECT images are usually integer gray values, and all results must be rounded to the nearest integer value. Therefore, when the strict monotonic condition is not satisfied, the method of finding the closest integer match is used to solve the problem of non-unique inverse transformation (10). **Figure 2** is the effect of histogram equalization on the improvement of SPECT image quality and the RGB color distribution diagram of the image before and after adjustment.

From the color distribution map before and after processing, it can be seen that in the RGB color distribution map before processing, the distribution ranges of the three colors are very narrow and concentrated in areas with higher gray levels. In the processed RGB color distribution diagram, each channel is more prominent except for the number of individual gray values, and the entire gray-value distribution is approximately uniform. It shows that after the SPECT image is equalized, the image becomes clear and the gray level of each pixel is reduced, but the distribution is more uniform and the contrast is higher.

#### 2.2.2 GrabCut

**Figure 3:**
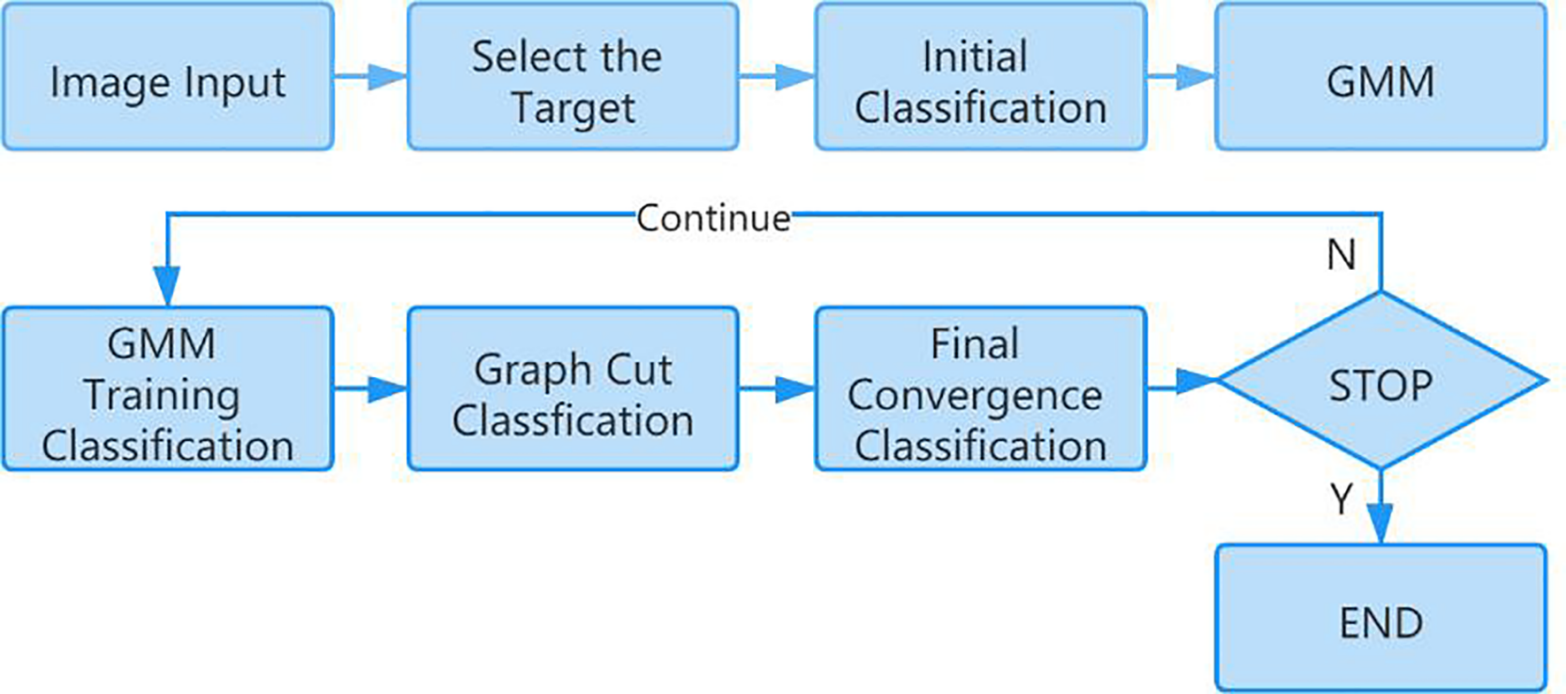
The calculation process of the GrabCut algorithm.

In this paper, the GrabCut algorithm is used to segment the human thyroid bed area from the image to reduce the interference caused by background noise (11, 12). GrabCut is a man-machine interactive image segmentation method. It uses the texture color information and boundary contrast information in the image, and only a small amount of user interaction can get a better segmentation result. GrabCut uses the RGB three-channel image as the input image and takes the RGB three-channel Gaussian mixture model GMM as the target and background model. Through continuous segmentation estimation and model parameter learning interactive iterations, the energy is minimized. The algorithm flow is mainly divided into two steps. The first step is initialization. Use a box to select the target in the input image. All the pixels outside the box are initialized to 0 as background pixels, and all the pixels in the box are initialized to 1 as possible target pixels. Then, the Gaussian mixture model of the target and background is estimated by pixel values. The target and background pixels are clustered into K classes through K-means clustering to obtain K Gaussian models. Each Gaussian mixture model calculates the mean and covariance of each Gaussian component through pixel samples. The second step is iterative minimization. The pixel value is substituted into the Gaussian component in the Gaussian mixture model of the result of the first step, the probability of each component is compared, and the pixel is assigned to the Gaussian component with the highest probability according to the result. **Figure 3** shows the calculation process of the GrabCut algorithm.

**Figure 4:**
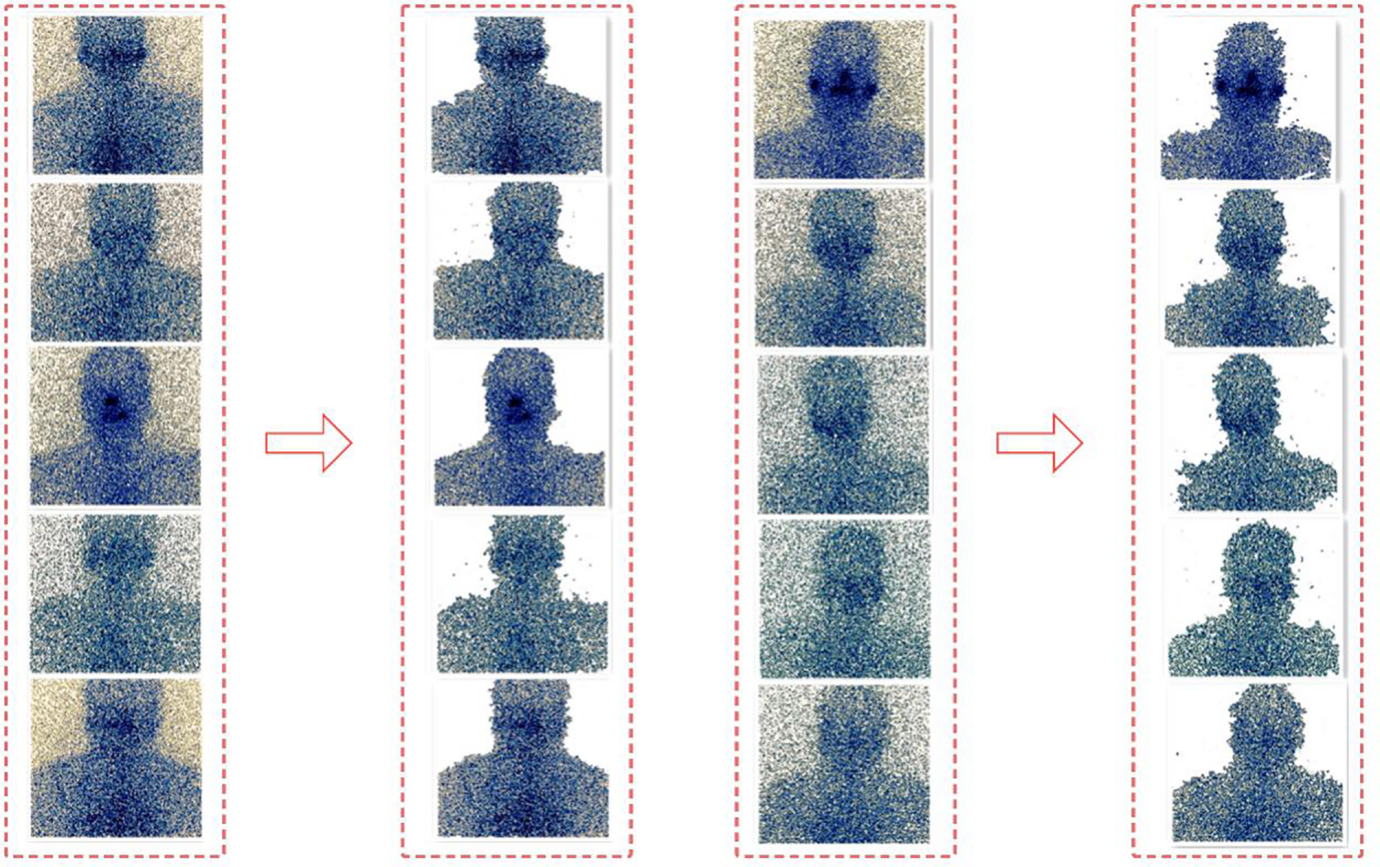
The segmentation effect of Grabcut on the SPECT.

Repeat the above two steps. In the iterative process, each pixel belongs to the background pixel or the target pixel will continue to change, and the energy function will continue to decrease to ensure that it can eventually converge (13). **Figure 4** shows the segmentation effect of GrabCut on the SPECT image.

### 2.3 Deep Residual Learning ResNet-18 Model

**Figure 5:**
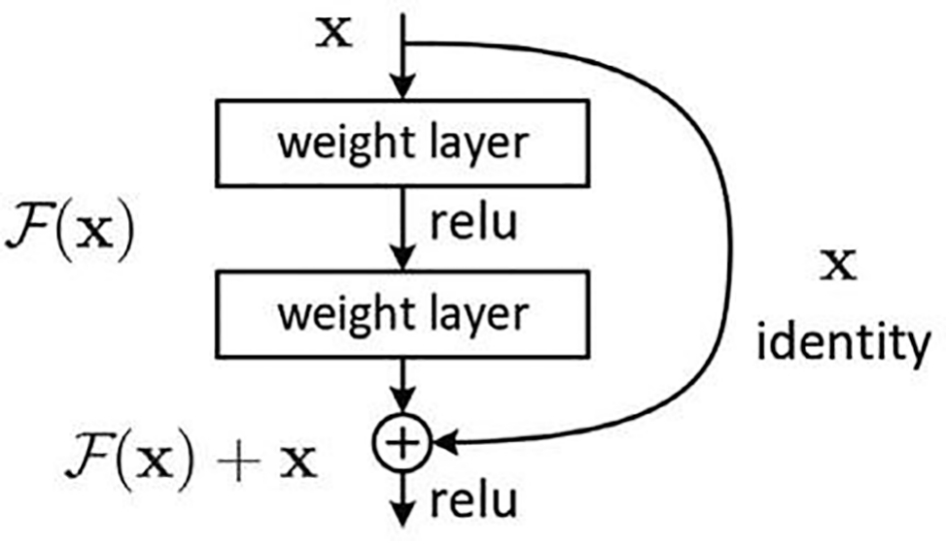
The basic structure of the Resnet.

The deep residual learning network was developed by researchers from Microsoft Research, and they won the first place in all the tracks in the ImageNet and COCO (14, 15) 2015 competitions. The difficulty of training deep networks is related to the back propagation of gradients. The deeper the network layer, the more difficult it is to update the gradient of the lower layer (the first layer). Therefore, the deep architecture cannot actually update these layers. ResNet developers think of the concept of residual representation commonly used in the computer field and apply it to the construction of ordinary CNN models. On the basis of ordinary networks, they form a new connection by adding a jump connection between each two layers of networks. The module is called the residual block. The author proves through experiments that ResNet can easily surpass the depth of traditional CNN through residual blocks and has a faster learning convergence speed and a more significant classification effect. The basic structure of the residual block is shown in **Figure 5**.

**Figure 6:**
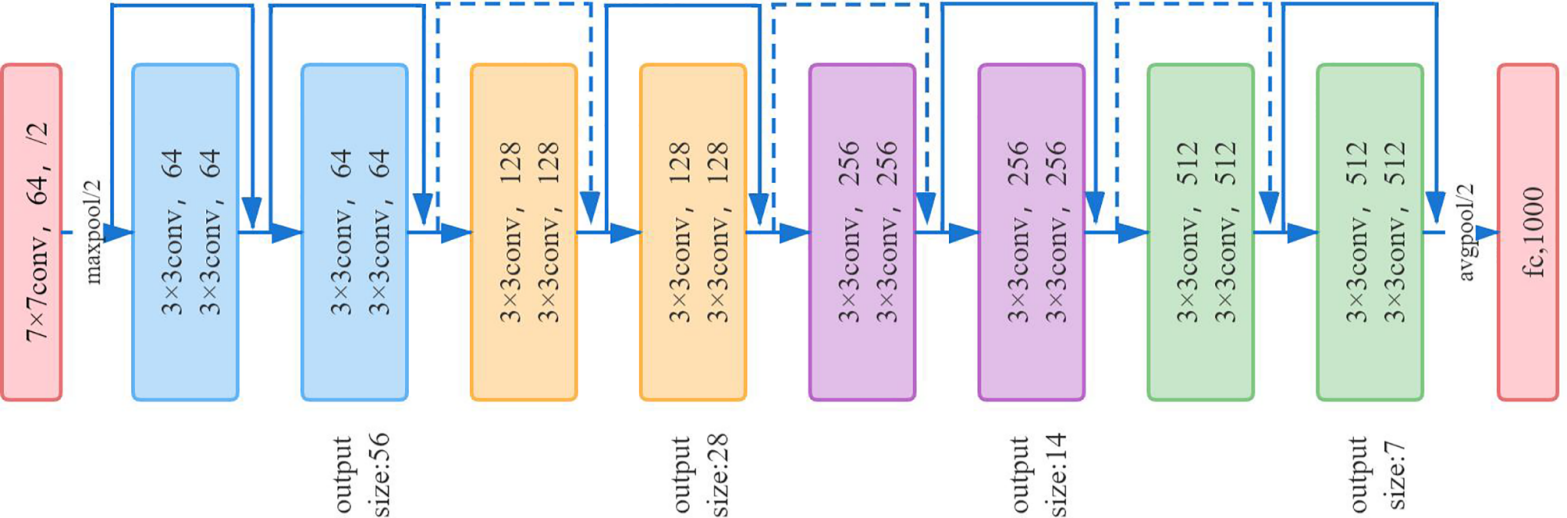
The network architecture of Resnet18.

ResNet-18 (16) has eight residual blocks; each block includes two convolutional layers, batch normalization layer and ReLU activation function. The output of each block is merged with its own input. Each convolutional layer in the residual block uses a 3 × 3 convolution kernel. In the first layer of the network, ResNet uses a 7 × 7 convolution with a step size of 2 to downsample the input and pooling layers. The last layer is the average pooling layer, which creates 1,000 feature maps based on ImageNet data. The result will be a 1,000-dimensional vector, which is then directly sent to the Softmax layer for classification. The network architecture of ResNet-18 is shown in **Figure 6**.

Due to the particularity of SPECT images, it is difficult for us to obtain large training samples as support to train convolutional neural network models from scratch, and it is very difficult to train deep learning models on a small amount of data. Therefore, we use a pretrained convolutional neural network to fine-tune the method to deal with this problem. In the classification task, the convolution part of the deep-learning model usually serves as the task of feature extraction, which can be transferred to related but different classification tasks by means of transfer learning. Although ultrasound images are very different from natural images, their perception of features is the same. The more samples you train, the more common the features will be. Fine-tuning is the process of adapting the weights of pretrained CNN to different data sets by using backpropagation. Fine-tuning a pretrained network is usually much faster and easier than training a CNN or neural network from scratch (17–19). We first pretrain ResNet-18 on the ImageNet dataset containing 1,000 types of natural images, so that it can learn various common image features and obtain a set of powerful parameters for initialization. Subsequently, we transfer the learned features and parameters from the general domain to the classification problem of SPECT images. The last fully connected layer of the original ResNet-18 outputs 1,000 categories of the ImageNet dataset. We fine-tune ResNet-18 by initializing a new fully connected layer, training on new SPECT image data, and learning and updating the parameters and weights of the fully connected layer through the backpropagation algorithm, so that the model only outputs two classes. That is, there is or no thyroid tissue remaining in the body.

**Figure 7:**
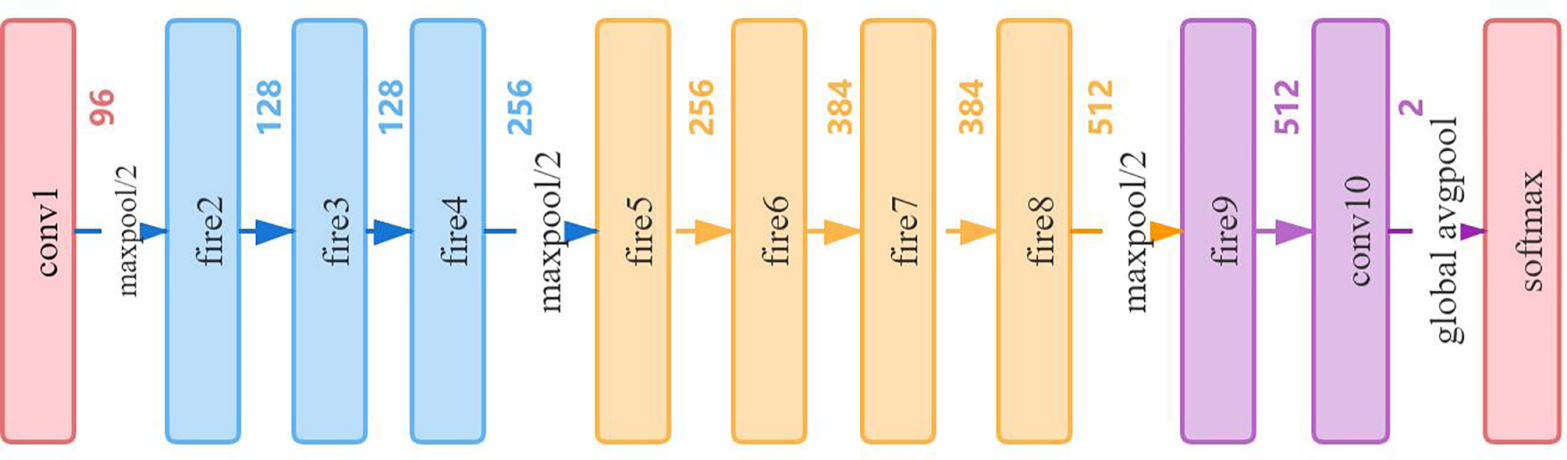
The network architecture of SqueezeNet.

Deep learning is developing rapidly in the field of computer vision; sophisticated and excellent advanced models have sprung up like mushrooms. They have achieved amazing accuracy in all major lists. However, limited by the actual application scenarios and resource equipment of the hospital, it is difficult to show the power of these complex models. Therefore, in real scenarios, lightweight models are more popular with people, and the actual application range is wider. Therefore, we decided to use the same fine-tuning technique to compare the proposed network architecture with the widely used lightweight networks SqueezeNet (20, 21) and ShuffleNetV2 (22, 23). **Table 2** shows the network architecture of ShuffleNetv2, and **Figure 7** shows the network architecture of SqueezeNet.

**Table 2:** Overall architecture of ShuffleNetv2.

Layer	Output size	Ksize	Stride	Repeat	Output channels			
					0.5×	1×	1.5×	2.5×
**Image**	**224 × 224**				**3**	**3**	**3**	**3**
**Conv1 MaxPool**	**112 × 112**	**3 × 3**	**2**	**1**	**24**	**24**	**24**	**24**
	**56 × 56**	**3 × 3**	**2**					
**Stage2**	**28 × 28**		**2**	**1**	**48**	**116**	**176**	**244**
	**28 × 28**		**1**	**3**				
**Stage3**	**14 × 14**		**2**	**1**	**96**	**232**	**352**	**488**
	**14 × 14**		**1**	**7**				
**Stage4**	**7 × 7**		**2**	**1**	**192**	**464**	**704**	**976**
	**7 × 7**		**1**	**3**				
**Conv5**	**7 × 7**	**1 × 1**	**1**	**1**	**1024**	**1024**	**1024**	**2048**
**GlobalPool**	**1 × 1**	**7 × 7**						
**FC**					**1,000**	**1,000**	**1,000**	**1,000**

### 2.4 Model Evaluation Index

In this paper, classification accuracy, sensitivity (recall rate), specificity, precision, F1-Score, and area under the receiver operating characteristic curve (AUC) are used to evaluate the performance of the two classification models. The accuracy rate represents the correct diagnosis rate of whether thyroid tissue remains. Sensitivity refers to the percentage of non-residue-type SPECT images correctly diagnosed as non-residue. Specificity refers to the percentage of SPECT images with residual types that are correctly classified as having residuals. The precision rate refers to the ratio of images that are predicted to have residual images that are actually residual images. The F1-Score indicator combines the results of precision and recall. The value range of F1-Score is from 0 to 1. 1 represents the best output of the model, and 0 represents the worst output of the model. The ROC curve is a tool to measure the balance between finding true positives and avoiding false positives. The AUC value is within the interval of [0.5, 1], indicating the possibility of correctly predicting whether the thyroid tissue remains in the body. A good classifier has an AUC close to 1.

## 3 Results

### 3.1 Dataset Division

Due to the small data set, in the case of insufficient sample size, in order to make full use of the data set to test the effect of the model, this paper takes a random grouping experiment (24, 25); a total of 10 experiments have been carried out. In each randomized experiment, the various images in the data set are randomly divided at a ratio of 3:1. The training set contains 335 images, including 260 images of residual type and 75 images of non-residual type. The test set contains 111 images, of which 86 are residual types and 25 are non-residual types. The data division is shown in **Table 3**.

**Table 3:** Number of images in the training set and test set.

	Total	Residual	Non-residual
**Training set**	**335**	**260**	**75**
**Testing set**	**111**	**86**	**25**

### 3.2 Fine-Tuning ResNet-18

**Figure 8:**
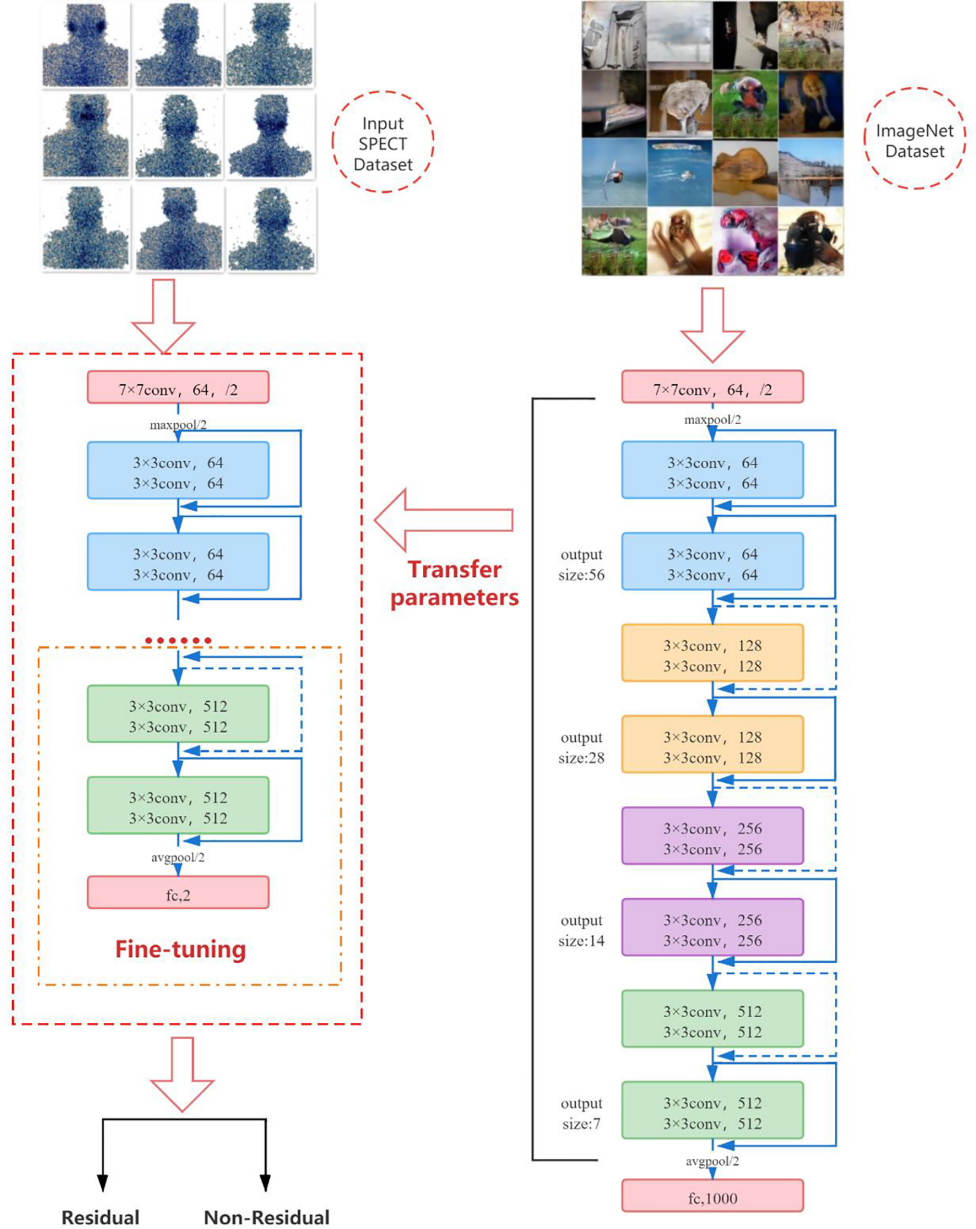
The fine-tuning process of the model.

The pretrained ResNet-18 model has been trained on the ImageNet dataset, and it contains the weights and biases that represent the features of the ImageNet dataset. Therefore, the pretrained model has learned common features including curves and edges, and these features can also be used in our classification problems. In this part of the work, we transfer the knowledge obtained from the ImageNet dataset from the pretrained model to another model and modify the final fully connected layer of the pretrained ResNet-18 to adapt ResNet-18 to our SPECT dataset. **Figure 8** shows the fine-tuning process of the model.

In order to obtain the best learning performance, it is very important to set appropriate parameter values of the backpropagation algorithm. Learning rate, momentum rate, and activation function are the main parameters that affect the learning performance of DCNN in the backpropagation algorithm. However, there are no clear guidelines for the selection of the best values of these parameters. The only way to check which parameters perform better is through multiple trials and errors. A higher learning rate value often leads to overfitting, while a lower learning rate value leads to limited error changes in different periods. In our experiments, the learning rate is chosen to be 10^–5^ through the experience of controlling the verification error in the fine-tuning process, so as to smoothly adapt to the new task without severely destroying the acquired knowledge. In the process of training and verification, the optimization algorithm Adam (26), which is an adaptive learning rate, is selected for parameter optimization. Compared with other optimization functions, Adam can calculate different adaptive learning rates for different parameters, and the memory requirements are relatively small. Moreover, the weight attenuation coefficient is 10^–5^. When the classifier output layer processes two classes, Softmax is the most appropriate as the activation function.

**Figure 9:**
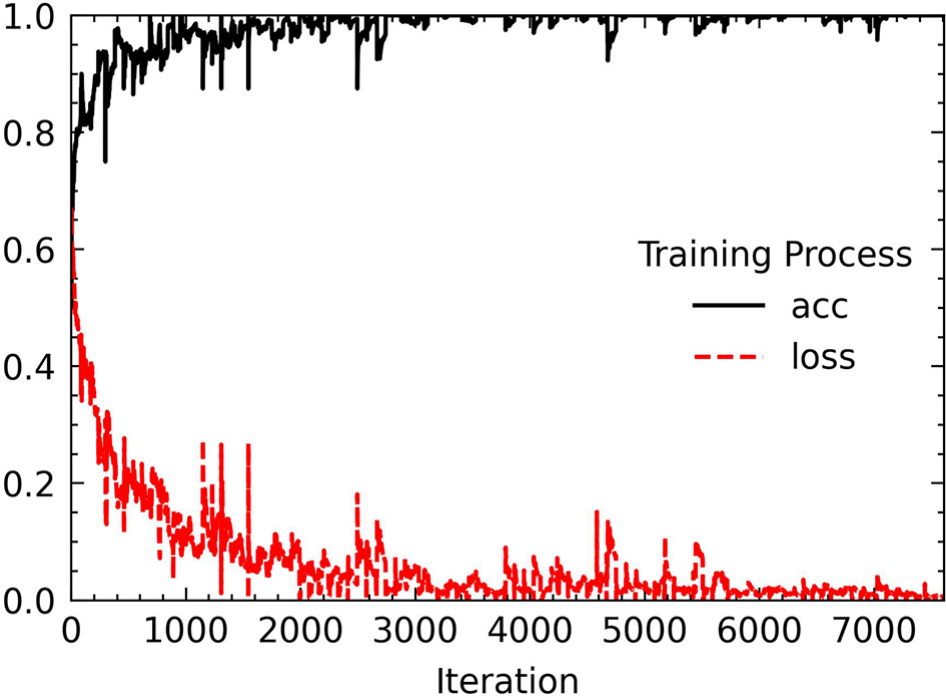
The training process of the proposed model.

Before putting the image data into network training, we also performed some processing on the data set. The first is to deal with the problem of class imbalance. We use the WeightedRandomSampler sampler under the PyTorch framework to perform weight sampling and use the idea of sampling from a set of samples based on polynomial distribution to assign weights to samples of different categories. Finally, a corresponding weight is added to each sample, and the number of images of different categories can be balanced during sampling. Then, each image put into training is sized to 224 × 224 for ResNet-18 training, and finally the image channels are standardized with the aim to prevent the convergence of too large numerical effects of the results. Standardization can accelerate the speed of convergence and to a certain extent can also improve the accuracy of convergence. We tried to increase the number of epochs from 30. Starting from 50 epochs, the accuracy of the model has not been greatly improved. When the epoch is set to 80, the model performance is the most stable and the loss curve is the most stable. The changes in accuracy and loss during the training process are shown in **Figure 9**.

### 3.3 Comparison of Diagnosis Performance of Different CAD Systems

All experiments use the same hardware configuration. The model used in this article has the advantage of a small amount of training parameters. The experiment can be performed on a personal computer device. All experiments are performed on the computer configured with Intel Core i7-7700 3.60 GHz CPU, 8 GB memory, and Windows 10 as the operating system. To assess the statistical significance of the performance differences, the non-parametric Mann–Whitney U test was performed for the values of each evaluation index. Areas under the ROC curve between each model were compared using the method of DeLong et al.

**Figure 10:**
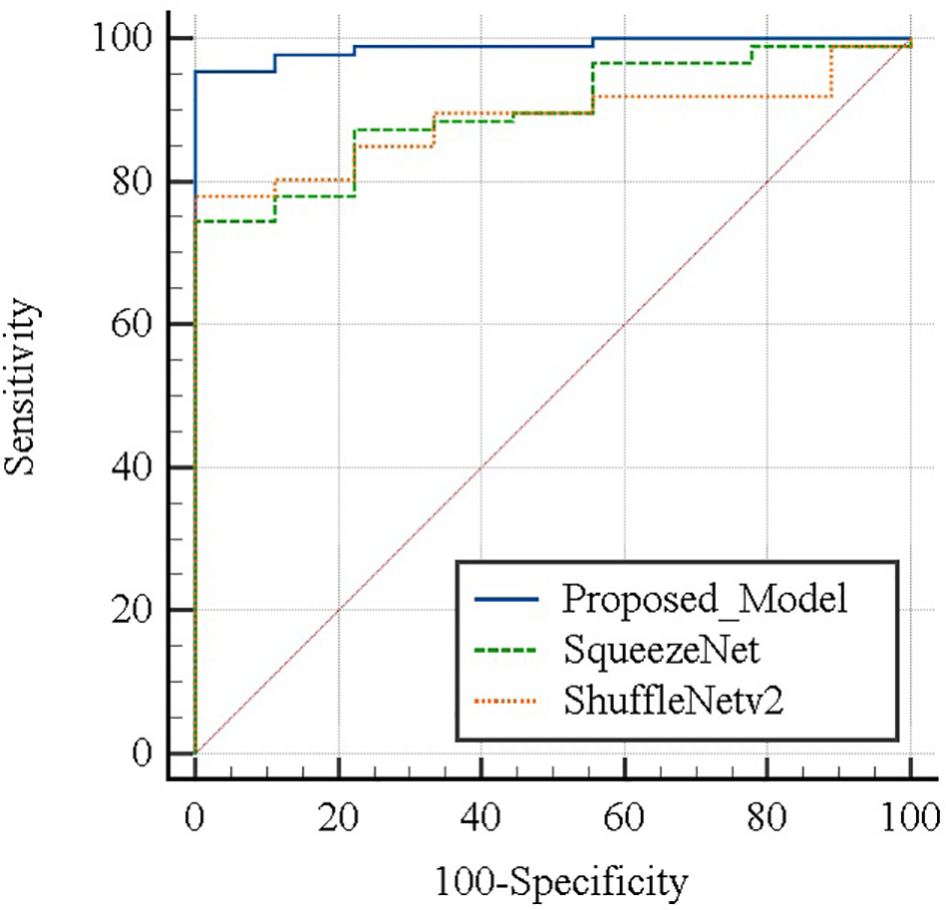
Areas under the ROC curve between each model were compared using the method of DeLong et al.

**Table 4** summarizes the performance of the proposed model in this article and the two lightweight models that are widely used in the diagnosis of residual thyroid tissue. Specifically, the classification accuracy of the proposed model in this paper is 96.69% ± 1.27%, the sensitivity is 94.75% ± 1.53%, the specificity is 99.6% ± 1.26%, the precision is 99.96% ± 0.09%, and the F1-Score is 97.55% ± 0.90%. Comparing the proposed model with the other two models, respectively, the accuracy and sensitivity of the proposed model and the other two models are statistically significant (p < 0.05), indicating that the proposed model has a better ability to diagnose residual thyroid tissue. There is no significant difference between the proposed model and the other two models in terms of specificity and sensitivity (p > 0.05). **Figure 10** shows the ROC curve of the proposed model and two lightweight models when judging whether thyroid tissue remains. The area under the curve values of the proposed model, SqueezeNet, and ShuffleNetv2 are 0.988 (95% CI, 0.941–1.000), 0.898 (95% CI, 0.819–0.951) (p = 0.0257), and 0.885 (95% CI, 0.803–0.941) (p = 0.0057), indicating that the accuracy of the proposed model is higher than that of SqueezeNet and ShuffleNetv2. These results mean that almost all SPECT images can be correctly classified by ResNet-18. At the same time, it is proved that fine-tuning the existing DCNN to generate a deep learning network in a specific field can effectively extract the high-level features of the SPECT image of the thyroid bed area and perform high-precision classification of whether there is residual thyroid tissue in the body after total thyroidectomy of thyroid cancer patients.

**Table 4:** Performance of each model in 10 randomized grouping experiments.

	Proposed model	SqueezeNet	p value	ShuffleNetv2	p value
ACC (%)	96.69 (1.27)	90.26 (0.98)	1.107e^−5^	86.39 (1.47)	1.111e^−5^
SEN (%)	94.75 (1.53)	88.66 (1.89)	1.111*e* ^−5^	81.7 (1.99)	1.119e^−5^
SPE (%)	99.6 (1.26)	98.8 (1.93)	0.168	98.4 (2.80)	0.297
PPV (%)	99.96 (0.09)	99.89 (0.17)	0.148	99.85 (0.24)	0.246
F1 score (%)	97.55 (0.90)	92.73 (0.75)	4.553e^−6^	89.18 (0.70)	1.021e^−5^

Numbers format: mean value (standard deviation).

## 4 Discussion

Computer-aided diagnosis (CAD) is a computerized technology that can be used as a second objective and accurate opinion. It can improve the accuracy and consistency of radiological diagnosis, reduce the reading time of radiological examination, and help radiologists perform interpretation and diagnosis of medical images (27). In recent years, computer-aided diagnosis (CAD) based on medical images, as a method that can effectively eliminate operation dependence and improve the accuracy of diagnosis, has performed very well in many disease diagnosis studies. CAD usually consists of three main steps: preprocessing, feature extraction, and classification. Generally speaking, preprocessing includes image denoising, contrast enhancement, edge enhancement, and segmentation. These steps not only are cumbersome but also have a great impact on subsequent processing. Extracting image features can be divided into morphological features and texture features extracted by machine learning models or using convolutional neural networks to extract feature maps. The classifier can be a support vector machine (SVM), k-nearest neighbor, AdaBoost, Gaussian mixture model, probabilistic neural network, decision tree, random forest, or Softmax. For example, Zhang et al. (28) established a CAD model on the ultrasound data set of 303 patients with thyroid cancer to classify and predict different types of malignant nodules. The specificity reached 86% and was no different from that of a senior radiologist. The sensitivity and accuracy of 78.6% and 90.7% are lower than those of senior radiologists, but they can significantly improve the diagnostic level of inexperienced radiologists. Ali et al. (29) established a deep artificial neural network model for lung cancer prediction based on 888 CT images of lung nodules from the LIDC/IDRI database. The accuracy was 99.1%, the sensitivity was 99.2%, and the specificity was 99.1%. Peng et al. (30) developed a deep learning artificial intelligence model (ThyNet) on thyroid nodule ultrasound images of 8,339 patients to distinguish malignant and benign thyroid nodules, achieving a significantly higher AUC (0.922 *vs*. 0.839) than radiologists and effectively reducing the number of unnecessary fine needle punctures in a simulated scenario. Fujisawa et al. (31) established a deep convolutional neural network model (DCNN) on the clinical image data set of 1,842 skin tumor patients from the University of Tsukuba Hospital to classify and diagnose 14 diseases. DCNN’s classification of skin tumor images reached 92.4%. The accuracy rate is higher than that of the committee-certified dermatologist (85.3%). Shakarami et al. (32) proposed a computer-aided diagnosis system COV-CAD to diagnose COVID-19 disease through lung images. The system uses fine-tuned AlexNet-CNN to extract features and uses a majority voting method to integrate multiple classifiers for final diagnosis; the accuracy rates of CT and X-ray data sets are 93.20% and 99.38%, respectively. Deepak et al. (33) studied the classification of brain tumors. Using the concept of deep transfer learning, pretrained GoogLeNet is used to extract features from brain MRI images, and a validated classifier model is integrated to classify the extracted features to distinguish gliomas, meningiomas, and pituitary tumors. A 98% classification accuracy rate was achieved on the MRI data set from figshare. Liu et al. (34) established a computer-aided diagnosis system for thyroid diseases based on the SPECT image data set to classify and predict hyperthyroidism, hypothyroidism, and normal and evaluated the classification effects of four CNN models. Among them, the VGG16 model has the best performance, with an accuracy rate of 96.2% and an AUC of 99.6%. Ma et al. (35) proposed a computer-aided diagnosis method based on the convolutional neural network (CNN) in 2018 and developed an improved CNN method with enhanced structure for deep learning to improve the performance of CNN. The proposed CNN method obtains the best accuracy and smaller error in the confusion matrix, can maintain performance under different iteration times, and has superior performance in the diagnosis of thyroid diseases. Furthermore, the DenseNet network was used to establish the diagnosis model of thyroid disease based on SPECT images in 2019, and its structure and training methods were improved on the basis of the traditional DenseNet network structure, which greatly improves the diagnosis effect of Graves’ disease, Hashimoto disease, and subacute thyroiditis (36).

In recent years, treatment strategies for thyroid cancer all over the world have the defect of overtreatment. In the diagnosis process of the doctor based on the SPECT image to determine whether there is thyroid tissue remaining in the body of a patient with thyroid cancer after ablation, in order to ensure that the suspected remaining patient is not at risk of recurrence, a certain dose of ^131^I will be given to the patient to remove these possible remaining thyroid tissue. Therefore, it is found that while doctors are making long-term plans for the patient’s physical condition, it is also difficult to avoid the occurrence of overtreatment problems. In this regard, we can pay attention to the specificity of the model, and we can find that the three models can reach more than 98% at the same time, which means that patients with no residual thyroid tissue in the body have a 98% chance of being correctly diagnosed. In addition, the precision rate is also an indicator worth noting. The precision of the three models is greater than 99%, which means that all patients with residuals predicted by the model are real patients with residuals. In other words, it represents that the probability of a patient with no residues in the body being misjudged as having residues is close to 0. Doctors can refer to the results of such classifications to reduce the dose of ^131^I medication for such patients, so as to reduce the harm to the patient’s body and reasonably reduce the occurrence of excessive treatment. On the other hand, we must also be able to identify as many patients as possible with real residual thyroid tissue in the body. In terms of sensitivity, the ResNet-18 model is significantly better than SqueezeNet and ShuffleNetv2. It can identify patients with residual thyroid tissue in the body with 96.69% accuracy, which shows that it can provide doctors with objectives and valuable second opinions and help them clarify the residual condition of thyroid tissue, reduce the workload, and avoid misdiagnosis due to visual fatigue.

Compared with the diagnosis research of some other diseases in the field of computer-aided diagnosis (19), the network model we proposed has the advantages of being light weight, having fewer training parameters, and having shorter training times while ensuring performance. It has the advantages of more convenience in experiments and applications. When our proposed method can be evaluated on a larger data set, it can be used as an auxiliary diagnostic tool in daily clinical practice without incurring additional installation costs. In addition, it can also save clinical diagnosis time. The model proposed in this paper takes an average of 20 s to make a diagnosis of an image. According to the nuclear medicine doctor of The Second Affiliated Hospital of Guangxi University of Science and Technology, it takes about 10 min to make a clinical diagnosis of an image. Therefore, we conservatively estimate that on each image, it can save clinicians at least 5 min on average. We recognize that this is an exploratory study, and our data set cannot adequately cover or represent the actual population base in clinical practice. Currently, we do not have enough data sets to verify the generalization ability of the model. With the continuous increase of clinical data, in the future work, we will more finely classify the residual thyroid tissue of patients with thyroid cancer after thyroid ablation, will predict the general situation of the residual thyroid tissue in the patient’s body, and can give more reference suggestions when the doctor chooses a more appropriate dose for the patient.

## 5 Conclusion

This paper is an exploratory work that applies deep learning to the classification of thyroid tissue residues in patients with thyroid cancer after ablation. We studied the feasibility of fine-tuning the deep convolutional neural network based on SPECT images to classify and predict the residual thyroid tissue in patients with thyroid cancer after ablation. We first use histogram equalization and GrabCut method to preprocess the SPECT image and propose a method of fine-tuning ResNet-18, which is applied to our data set through transfer learning. Finally, experiments were conducted on SPECT images of 446 patients and performance comparisons were made with the widely used lightweight network SqueezeNet and ShuffleNetV2 models. The accuracy, sensitivity, specificity, precision and F1-Score of the proposed model are 96.69%, 94.75%, 99.6%, 99.96%, and 97.55%, respectively, and the AUC is 0.988. Experiments have proved that the strategy of fine-tuning the pretraining DCNN model in this paper and learning the relevant features of image classification on the natural image data set is beneficial to improving the training process and can successfully transfer the knowledge and parameters obtained by pretraining to the task of thyroid tissue residual classification, The thyroid tissue residue classification system based on the deep residual network ResNet-18 proposed in this paper can be used as a computer-aided diagnosis method to improve the diagnosis of thyroid tissue residues in patients with thyroid cancer after ablation. While more accurately diagnosing patients with residual thyroid tissue in the body, we have tried our best to avoid the occurrence of overtreatment.

## Data Availability Statement

The raw data supporting the conclusions of this article will be made available by the authors, without undue reservation.

## Ethics Statement

The studies involving human participants were reviewed and approved by the Medical Ethics Committee of the Second Affiliated Hospital of Guangxi University of Science and Technology. Written informed consent for participation was not required for this study in accordance with the national legislation and the institutional requirements.

## Author Contributions

YG and JX were major contributors in writing the manuscript, have contributed equally to this work, and share first authorship. YG, JX, XL, XY, and WP conceived and designed the experiments. SM, DH, and MQ organized the database. YG and LZ did the literature research. All authors contributed to the manuscript revision and read and approved the submitted version.

## Funding

This work was supported by grants from the Young and Middle-aged Teachers’ Scientific Research Basic Ability Promotion Project of Guangxi Universities (2021KY0339), Doctoral program of Guangxi University of Science and Technology (21Z01 and 21Z24), and Self-funded Scientific Research Project of Guangxi Health Commission (Z20212280).

## Conflict of Interest

The authors declare that the research was conducted in the absence of any commercial or financial relationships that could be construed as a potential conflict of interest.

## Publisher’s Note

All claims expressed in this article are solely those of the authors and do not necessarily represent those of their affiliated organizations, or those of the publisher, the editors and the reviewers. Any product that may be evaluated in this article, or claim that may be made by its manufacturer, is not guaranteed or endorsed by the publisher.
